# Case Report: Severe *Listeria* Encephalitis With Complicated or Secondary Autoimmune Encephalitis and CNS Demyelinating Diseases

**DOI:** 10.3389/fpubh.2022.848868

**Published:** 2022-05-12

**Authors:** Xiaomin Zhang, Peng Feng, Pengfei Meng, Dongfang Li, Huizhong Gao, Yang Zhao, Jingjing Yuan, Yongqing Wang, Han Xia

**Affiliations:** ^1^Department of Neurology, The Second Hospital of Shanxi Medical University, Taiyuan, China; ^2^Acupuncture Hospital of Shanxi Province, Taiyuan, China; ^3^Hugobiotech Co., Ltd., Department of Scientific Affairs, Beijing, China

**Keywords:** severe *Listeria* encephalitis, complicated or secondary autoimmune encephalitis, CNS demyelinating diseases, metagenomic next-generation sequencing, pathogen detection

## Abstract

**Background:**

*Listeria monocytogenes* is an important food-borne bacterium. It rarely infects patients with complete immunity and causes meningocephalitis. Patients with severe *Listeria* encephalitis always experience a bad prognosis.

**Case Presentation:**

A 39-year-old male patient was admitted to our hospital due to fever for more than 10 days and disturbance of consciousness accompanied by convulsions for 2 days. Metagenomic next-generation sequencing (mNGS) results showed *L. monocytogenes* in both cerebrospinal fluid (CSF) and blood, indicating *L. monocytogenes* encephalitis. Autoimmune encephalitis and central nervous system (CNS) demyelinating autoantibodies in the CSF also showed positive results. The case was finally diagnosed as severe *Listeria* encephalitis with complicated or secondary autoimmune encephalitis and CNS demyelinating diseases.

**Conclusions:**

It is necessary to carry out infection and immunity screening in patients with severe encephalitis, especially for immunocompromised patients. mNGS plays a pivotal role in screening patients with severe and difficult neurological diseases.

## Introduction

*Listeria* was discovered at the dawn of the 20th century. There are a total of 7 bacterial genera, which are Gram-positive and facultatively anaerobic. This zoonotic pathogen most suitably grows in micro-oxygen environment containing carbon dioxide. *Listeria monocytogenes* is the only flora capable of causing human disease and is the cause of food-borne infection (1). This bacterium is able to grow and reproduce in a 4°C environment. Although mainly transmitting through frozen food, it can also enter the body through eyes, skin, and mucous membranes to cause infection (2). It is highly neurophilic (3), mostly in neonates, elderly, pregnant women, and immunologically compromised population such as those receiving immunosuppressants.

*L. monocytogenes* disease (LD) is caused by infection of *L. monocytogenes*. The pathogenesis may be that *L. monocytogenes* enters the blood circulation when the body's immunity is compromised to release hemolysin and listerin, causing damage to cerebrovascular endothelial cells and increased permeability of the blood-brain barrier. After infiltrating the CSF, *L. monocytogenes* proliferates and releases a large amount of hemolysin and listerin. The resulted subarachnoid inflammation and intracranial hypertension eventually lead to central nervous system injuries (4).

Patients with LD always experience no typical clinical symptoms or image manifestations, including high fever, severe headache, nausea and vomiting, diarrhea (5). However, *L. monocytogenes* can also cause serve illnesses, and the fatality rate is 24–62% (5). In recent years, the incidence of LD has gradually increased and caused a burden for public health (6).

Here we report a rare case with severe *Listeria* encephalitis with complicated or secondary autoimmune encephalitis and central nervous system (CNS) demyelinating diseases, which has never been reported before.

## Case Description

### Patient History

A 39-year-old male patient was admitted to the Department of Emergency Medicine, the Second Hospital of Shanxi Medical University on May 12th, 2020, due to chief complaints of fever for more than 10 days and disturbance of consciousness accompanied by convulsions for 2 days.

The patient developed intermittent fever before May 1st, 2020 (the specific time was unknown), the highest temperature was ~37.5°C, accompanied by chest tightness. The conditions were unresponsive to self-administered oral anti-inflammatory drugs. Diarrhea occurred on May 7th, and the conditions gradually deteriorated. On May 10th, the temperature elevated to 39°C, accompanied by headache, confusion, and inability to answer questions correctly.

The patient had a history of intermittent “low fever” for 7–8 years. Due to his suspected “rheumatoid arthritis,” the patient had taken oral medications (Chinese patent medicines and hormones, but the specific details were unknown) irregularly for many years. The patient had been married for 10 years without children. His wife was in good health and claimed that her reproductive system examination showed no abnormalities.

### Diagnosis and Treatment After Admission

When admission, the patient was admitted to the ICU of the Department of Neurology for further diagnosis and treatment.

Physical examinations on admission showed the body temperature at 40°C, pulse at 120 beats/min, respiration rate at 20 times/min, blood pressure at 120/70 mmHg, blood oxygen saturation at 96%, and assisted ventilation. Nervous system examinations indicated moderate coma. The pupils were equal in size and roundness with a diameter of ~0.3 cm, and the light reflex was normal. His muscular tone of four limbs slightly increased. Meningeal irritation signs were positive, and bilateral pathological signs were negative.

The patient was uncooperative with the rest physical examinations. Related biochemical and imaging examinations were also tested. Emergency blood gas analysis showed pH at 7.457, PCO_2_ at 28.9 mmHg, and PO_2_ at 97.7 mmHg. Routine blood test showed WBC counts at 12.34 × 10^9^/L, RBC counts at 4.30 × 10^12^/L, hemoglobin concentration at 118.0 g/L, platelet counts at 258.00 × 10^9^/L, and C-reactive protein at 277.20 mg/L. Head CT did not reveal any obvious abnormalities, while chest CT showed mild pneumonia of the lower lobes.

Initial treatments included ice blanket and ice cap for physical cooling, empirical antiviral and antibacterial therapies, intravenous administration of γ-globulin to improve immunity, elimination of autoimmune antibodies, rehydration, and other symptomatic and supportive treatment. At 23:18 of that night, the patient manifested sudden convulsions of the four limbs, loss of consciousness, lockjaw, and ocular gaze to the left. After intravenous injection of 10 mg diazepam, ocular gaze, limb stiffness, and convulsion stopped, but strong limb struggling occurred. Propofol was then administered for sedation purpose, followed by tracheal intubation, tracheotomy, and assisted ventilation. Subsequently, propofol combined with midazolam, meanwhile, antiviral, antibacterial, physical cooling, fluid rehydration, and other therapies were provided, but the temperature still elevated intermittently, up to 41°C.

Relevant laboratory examinations were completed: Blood coagulation series showed prothrombin time control at 13.50 S, prothrombin time measurement at 12.7 S, D-dimer at 1,892 ng/ml. Procalcitonin was tested at 3.53 ng/ml. ESR was at 120.00 mm/h. IgG was at 15.70 g/L, IgA was <0.0667 g/L, IgM at 0.53 g/L. *Mycobacterium tuberculosis* antibody test and sputum acid-fast bacillus smear showed negative. Blood biochemistry showed Urea at 7.62 mmol/L, creatinine at 75.43 μmol/L, potassium at 3.83 mmol/L, sodium at 143.00 mmol/L, chlorine at 112.00 mmol/L, creatine kinase isoenzyme MB at 0.42 ng/mL, myoglobin at 103.02 ng/mL, hypersensitive troponin at 0.02 ng/mL, B-type natriuretic peptide at 38.38 pg/ml, ALT at 23.10 U/L, AST at 48.20 U/L, albumin at 24.20 g/L, and LDL at 2.43 mmol/L. Thyroid function showed Serum T3 3.75 pmol/L, serum free T4 12.71 pmol/L, highly sensitive serum TSH 0.76 mIU/L; 8. Folic acid 21.08 nmol/L, vitamin B_12_ 252.00 pmol/L. CSF examination showed high pressure, WBC counts at 680 × 10^6^/L, microscopic multinuclear cells at 81%, microscopic lymphocytes at 19%, RBC counts 170 × 10^6^/L, γ-globulin test (+), glucose at 0.69 mmol/L (2.2–3.9), chlorine at 124 mmol/L, and CSF proteins at 3.28 g/L (0.08–0.43).

**Figure 1:**
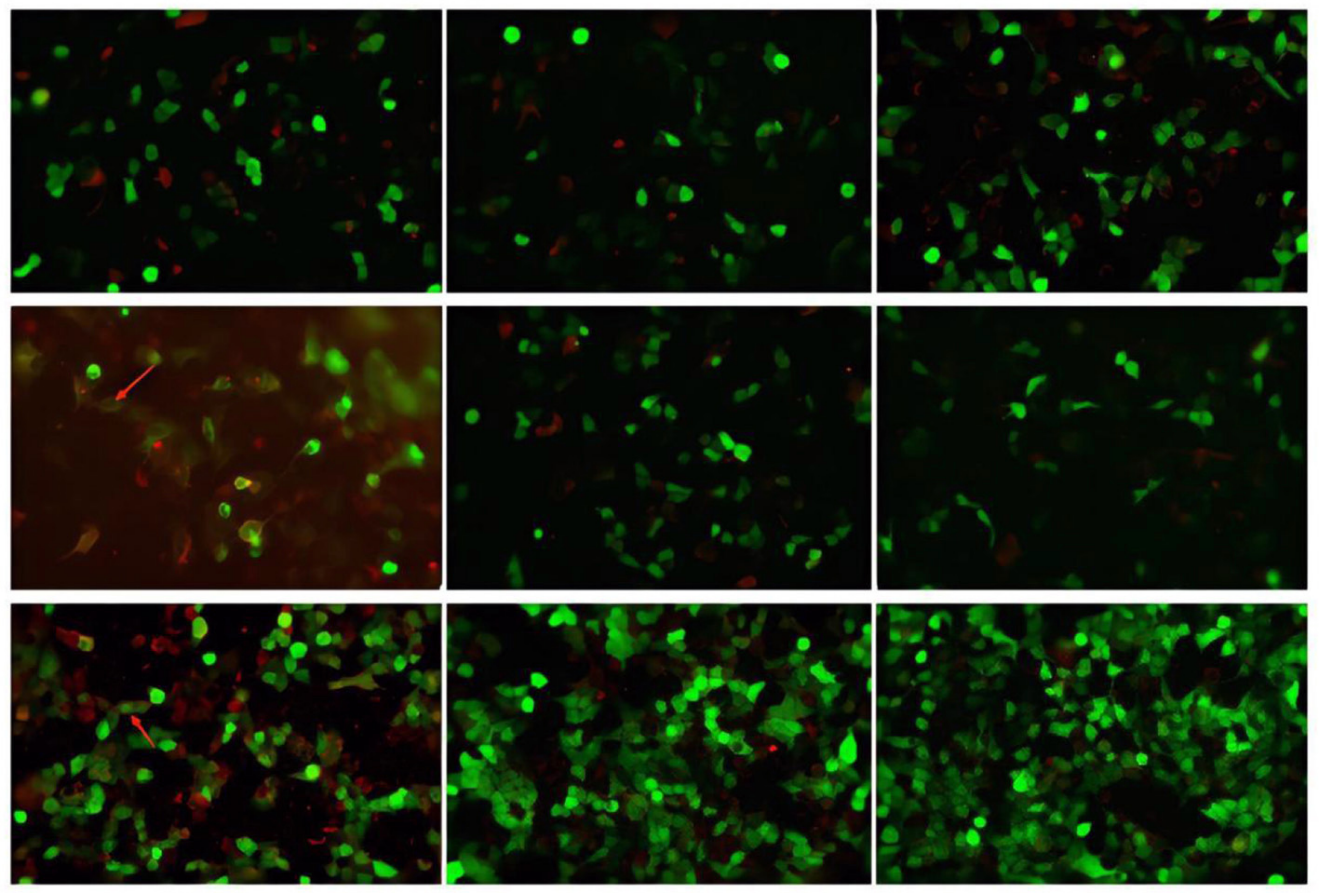
Autoimmune encephalitis autoantibody, paraneoplastic syndrome autoantibody, and central nervous system demyeling antibody results of CSF samples. The fluorescence result is positive/weakly positive and have been marked with arrows.

**Figure 2:**
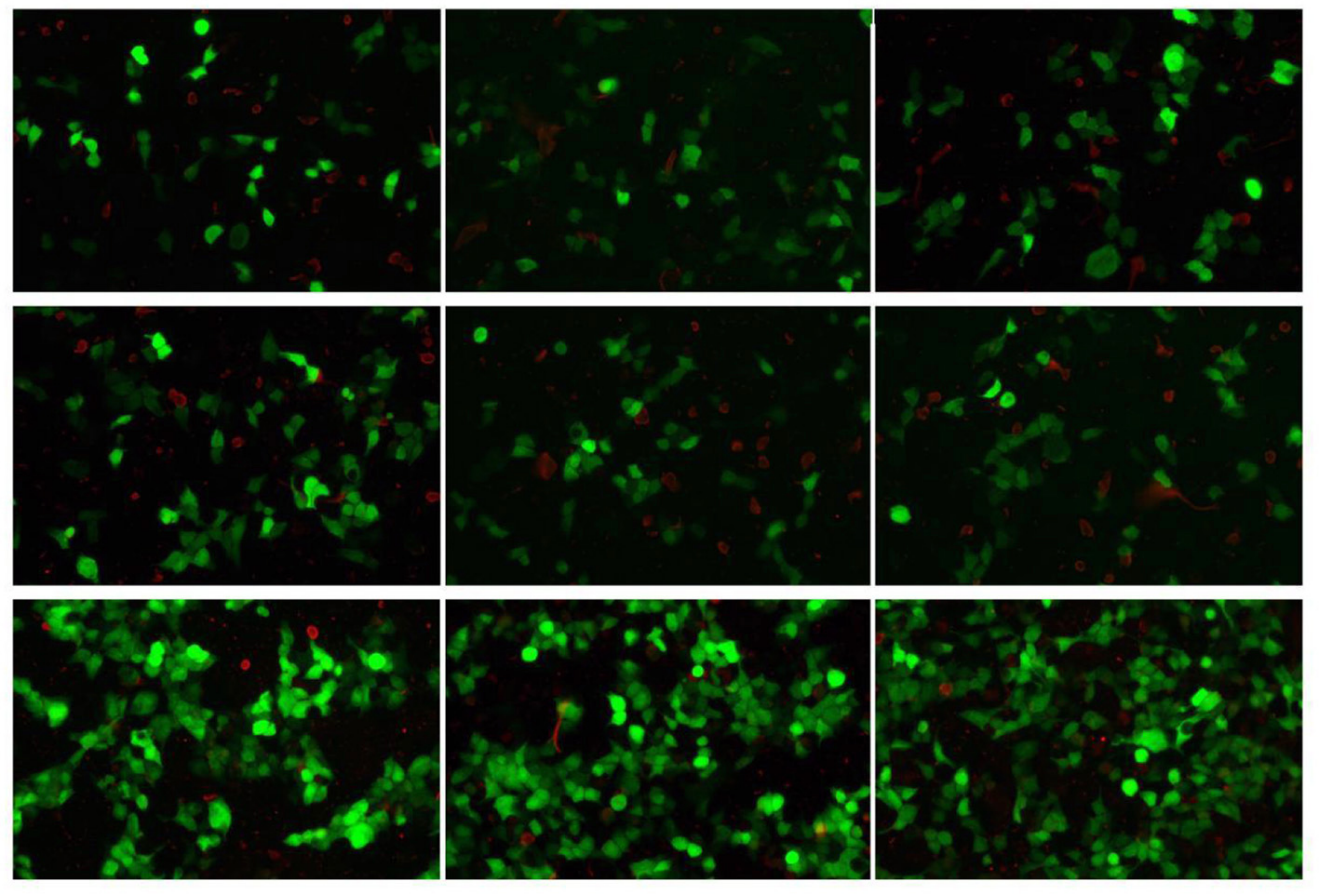
Autoimmune encephalitis autoantibody, paraneoplastic syndrome autoantibody, and central nervous system demyeling antibody results of blood samples. The fluorescence result was negative.

**Figure 3:**
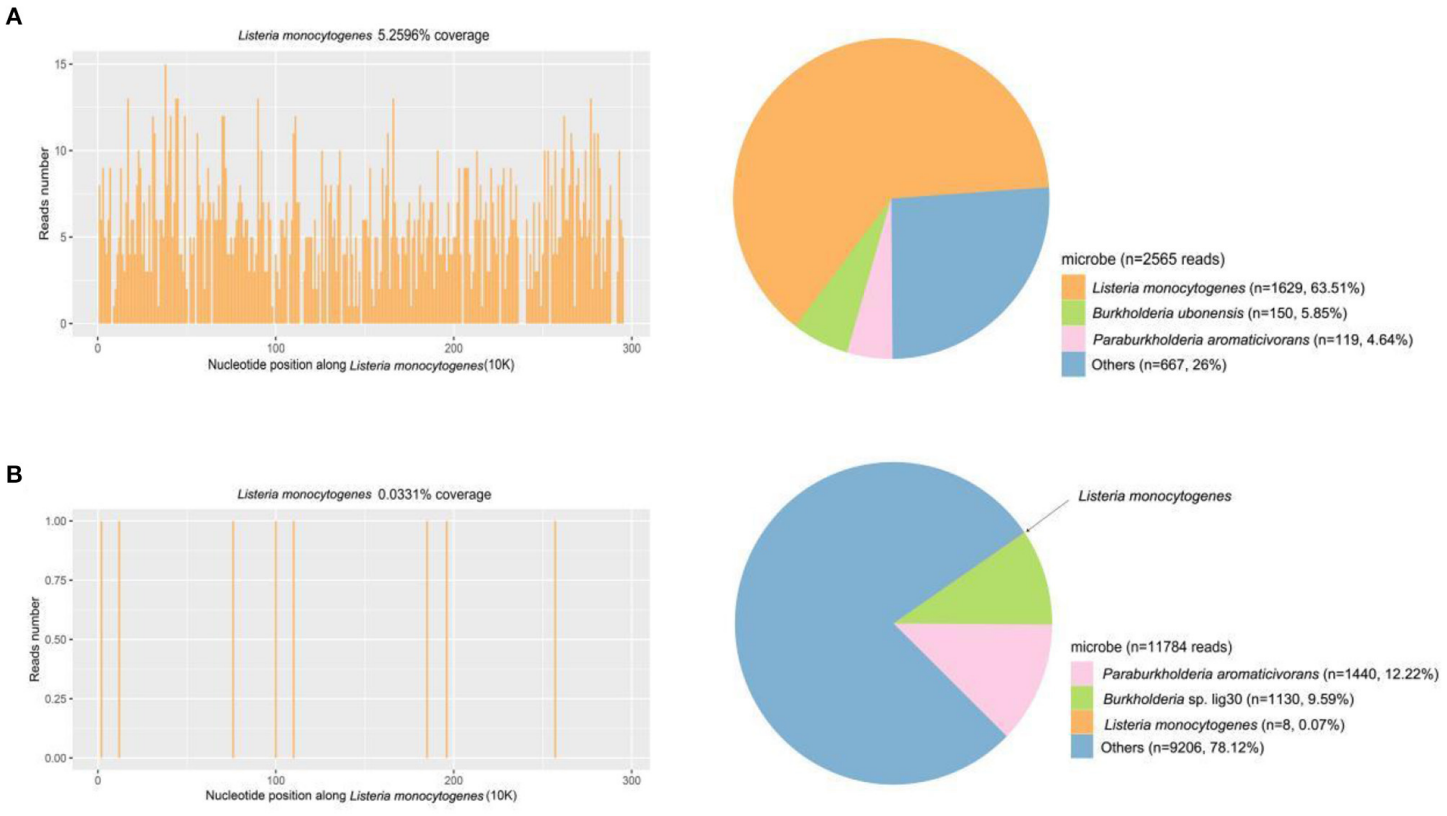
mNGS results of CSF and blood samples of the patient. **(A)** A total of 1,629 unique reads of *Listeria monocytogenes* were detected by CSF mNGS, with a coverage of 5.26%. **(B)** Eight unique reads of *Listeria monocytogenes* were detected by blood mNGS, with a coverage of 0.03%.

CSF virus series (human cytomegalovirus and EB virus) and ink staining (*Cryptococcus* and *Mycobacterium tuberculosis*) showed negative. CSF immunoglobulin showed IgG at 1250.00 mg/L, IgA at 0.91 mg/L, and IgM at 4.37 mg/L. Autoimmune encephalitis autoantibody, paraneoplastic syndrome autoantibody, and central nervous system demyeling antibody of CSF and blood were tested. The results of CSF were positive/weakly positive, while the blood results were all negative (Figures 1, 2). The CSF and blood samples of the patient were also sent for PACEseq metagenomic next-generation sequencing (mNGS) detection (Hugobiotech, Beijing, China). *L. monocytogenes* was identified in both CSF and blood, with the specific sequence number of 1,629 and 8, respectively (Figure 3).

Based on the patient's symptoms, extra information was added according to the inquiry into the medical history of family members. The patient usually did not pay much attention to oral hygiene. He brushed teeth irregularly, and often ate food directly out of the refrigerator. He felt oral discomfort 2 days before disease onset. The patient had repeatedly instilled tobramycin eye drops due to eye discomfort, and often squeezed multiple acne on the face by himself. The past medication history included the use of a large amount of oral drugs for the treatment of rheumatoid arthritis, without seeking formal medical treatments, instead, he often searched the Internet for medicines on his own and orally administered more than 10 drugs (including non-steroidal hormonal anti-inflammatory drugs). Therefore, this patient had a history of ingesting unclean food and facial infection, as well as the use of multiple drugs including glucocorticoids. This could lead to a weakened and compromised immunity, causing *L. monocytogenes* invasion into the central nervous system.

The patient was finally diagnosed as *Listeria* meningoencephalitis with secondary epilepsy and status epilepticus, bilateral pneumonia, and perhaps rheumatoid arthritis. Thus, adjusted treatments including ampicillin and rifampicin in combination with meropenem, linezolid, and acyclovir were given. Anti-epileptic therapy, dehydration to reduce cerebral edema, and intravenous infusion of globulin symptomatic and supportive treatments were also provided.

### Follow-Up Treatment and Outcome

After 10 days of treatments, the patient symptoms partially improved. The consciousness gradually regained. Seizures did not recur. His temperature gradually decreased. However, the patient's responsiveness was unsatisfactory. In the meantime, new problems emerged. Despite temperature decline, low fever persisted for a long time, fluctuating at 37.5–38.0°C, and ESR continued to be around 110 mm/h. Neurological examination showed suspicious paralysis of limbs and ophthalmoplegia: despite the consciousness, the patient was unable to speak due to tracheotomy or follow the sound, both eyes was able to move leftward, but the left eye outreached poorly, both pupils was equal in size and roundness with a diameter of 0.3 cm, light reflex was normal, muscle tone of the upper limbs slightly increased, while muscle tone of the lower limbs was basically normal, there was no voluntary movement of the four limbs even under strong stimulation, and bilateral pathological signs were negative. During treatment, the patient developed severe anemia, which met the indication for blood transfusion, unfortunately, repeated blood matching failed.

On May 25th, 500 mg methylprednisolone pulse therapy was added. On the next day, the patient's symptoms significantly relieved. Both hands could raise off the bed, and muscle contraction of both lower limbs was observed. During the following more than 10 days of treatments (the protocol was the same as above), the patient's symptoms gradually improved. The temperature decreased to normal. The patient regained consciousness and was able to speak. His muscle tone of four limbs reached level 4, and ophthalmoplegia gradually alleviated. Repeated examinations of CSF routine and biochemistry gradually returned normal. ESR gradually decreased to normal level.

**Figure 4:**
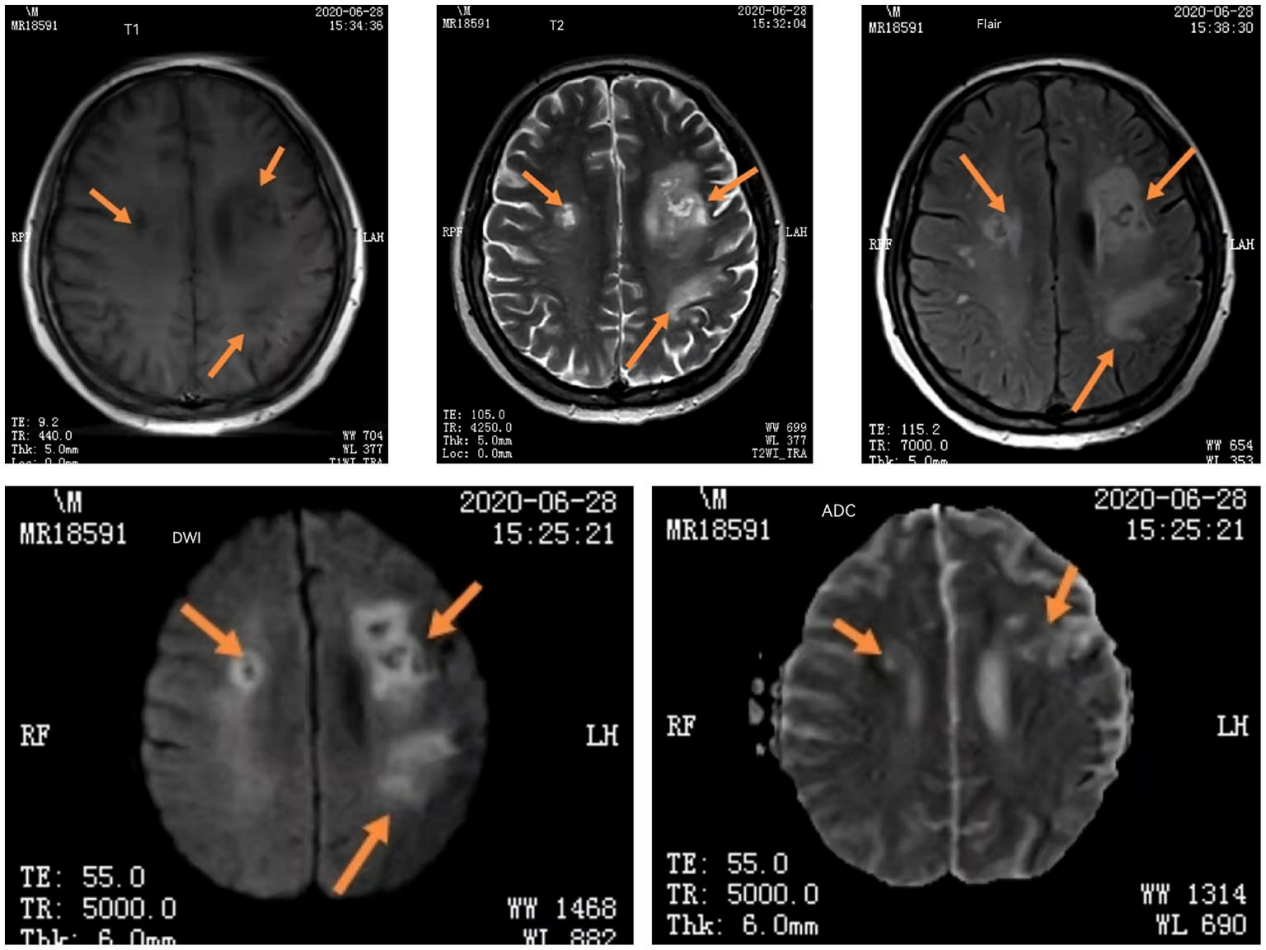
The iconography results of re-examinations of the patient. Head MRI (T1, T2, FLAIR, DWI, ADC) showed bilateral frontal and parietal cortex necrosis and meningoencephalitis. The arrows are showing changes in head MRI of bilateral frontal and parietal cortex necrosis and meningoencephalitis.

On June 28th, mNGS result reported the number of specific sequences of *L. monocytogenes* decreased to 136. Subsequent re-examinations of MRI (T1, T2, FLAIR, DWI, ADC) showed bilateral frontal and parietal cortex necrosis and meningoencephalitis (Figure 4). The patient was instructed to strengthen rehabilitation training. Reexamination of the CSF on July 12th reported mononuclear cell proliferation, *L. monocytogenes*, and autoimmune antibody (-). Since the follow-up to present, small oral dose of hormone was regularly administered, and the disease has not recurred.

## Discussion and Conclusions

*L. monocytogenes* can lead to significant intracranial hypertension, the CSF primarily manifests significant elevation of cell counts, mainly the percentage of multinuclear cells, and significant increase in proteins.

In this case, mNGS results played an important role in the diagnosis of pathogens. Previous studies have demonstrated that human cerebrospinal fluid does not have a microbiome but may contain potential pathogens (7, 8), making highly sensitive assays such as mNGS particularly suitable for the detection of cerebrospinal fluid samples. And, with further improvements in sensitivity due to continuous improvements in the removal of human contamination from sequencing results (9), its detection results will be increasingly worthy of clinical attention.

Currently, the clinical treatment of *Listeriosis* mainly relies on β-lactam antibiotics (ampicillin and penicillin). It is sensitive to sulfonamides, quinolones, and rifampin, among which, rifampicin can easily penetrate the blood-brain barrier and has a potent effect on these bacterium (10). Some literature pointed out that meropenem exhibited activity against *L. monocytogenes in vitro* (11), and there was a case report of successful cure with meropenem (12). This patient received meropenem immediately after admission. Once the diagnosis was confirmed, symptomatic and supportive treatments, including the addition of ampicillin and rifampicin, were given.

Considering that autoantibodies adsorbed on the surface of red blood cells may cause hemolytic anemia (13) and that 2–10% of male infertility is related to immune factors (14), although antimicrobial therapy did not reach the guideline recommendation of 21 days or longer (15), the patient was eventually treated with an experimental hormone pulse. The next day the patient's symptoms resolved rapidly. Therefore, the complicated or secondary autoimmune encephalitis and central nervous system demyelinating disease was confirmed.

Autoimmune encephalitis is an antibody-mediated injury and an inflammatory disease of the central nervous system (16). The most common clinical manifestations are multiple sclerosis and neuromyelitis optica, the latter is primarily related to AQP4-IgG (17). However, *Listeria* meningoencephalitis complicated with autoimmune encephalitis and central nervous demyelinating disease is clinically rare, and there is no relevant literature report yet.

By summarizing this case, we have realized the followings: Firstly, it is necessary to carry out infection and immunity screening in patients with severe encephalitis. Secondly, mNGS of CSF, with its high throughput and unbiased nature, has been widely used in the clinic (18) and is indeed an effective pathogen detection technique. Thirdly, this patient's symptoms quickly relieved after the use of hormone on the basis of anti-infection, However, the establishment of *Listeria* meningoencephalitis with complicated or secondary autoimmune encephalitis and central nervous system demyelinating diseases still requires us to constantly summarize our experience and to continue to explore in future clinical practice. Fourthly, understanding of the patient's medical history is the top priority for every physician. It is important to inquire the medical history repeatedly and carefully, including personal history and past history. In addition, building trust with patient's family members within a limited time is the key for effective communication.

## Data Availability Statement

The data presented in the study are deposited in the National Genomics Data Center (https://www.cncb.ac.cn/) repository, accession number PRJCA007732.

## Author Contributions

XZ and PF designed and drafted the paper. XZ, PF, PM, YZ, HG, DL, JY, and YW involved in the clinical care and management of the patients. HX analyzed the mNGS data. All authors approved the final manuscript as submitted and agree to be accountable for all aspects of the work.

## Funding

The research has been supported by the Science and Technology Project of Xi'an (No. 21RGSF0013).

## Conflict of Interest

HX is employed by Hugobiotech Co., Ltd. The remaining authors declare that the research was conducted in the absence of any commercial or financial relationships that could be construed as a potential conflict of interest.

## Publisher's Note

All claims expressed in this article are solely those of the authors and do not necessarily represent those of their affiliated organizations, or those of the publisher, the editors and the reviewers. Any product that may be evaluated in this article, or claim that may be made by its manufacturer, is not guaranteed or endorsed by the publisher.
